# Assessing mortality risk in pulmonary tuberculosis and severe malnutrition: development of the IIR marker via artificial intelligence

**DOI:** 10.1038/s41598-026-39487-3

**Published:** 2026-02-19

**Authors:** Dumitru Rădulescu, Costin-Teodor Streba, Emil-Tiberius Traşcă, Patricia-Mihaela Rădulescu, Liliana Streba, Iulian-Laurenţiu Buican, Cristina Călăraşu

**Affiliations:** 1 Department of Surgery, University of Medicine and Pharmacy of Craiova, Craiova, Romania; 2 Department of Pulmonology, University of Medicine and Pharmacy of Craiova, Craiova, Romania; 3 Department of Oncology, University of Medicine and Pharmacy of Craiova, Craiova, Romania; 4 U.M.F. Doctoral School Craiova, University of Medicine and Pharmacy of Craiova, Craiova, Romania

**Keywords:** Tuberculosis, Malnutrition, Mortality prediction, Artificial intelligence, Machine learning, Immuno-inflammatory ratio (IIR), Medical research, Biomarkers, Predictive markers, Prognostic markers

## Abstract

The early identification of mortality risk in patients with tuberculosis (TB) and severe malnutrition (BMI <16 kg/m^2^) is critical for optimizing clinical outcomes. In this three-year ambispective study (October 1, 2021–September 30, 2024), conducted at Leamna Hospital, a reference center for the Oltenia Region, Romania, 216 patients with pulmonary tuberculosis were selected from a total of 3,547 TB cases for analysis. We assessed all-cause in-hospital mortality during the index admission only (from admission to discharge); deaths after discharge or during subsequent admissions were excluded, patients transferred without cross-facility linkage were right-censored at transfer, and all analyses used baseline hematological and biochemical parameters obtained before initiation of any treatment. We developed and validated the Immuno-Inflammatory Ratio (IIR), a novel machine-learning–assisted biomarker integrating neutrophils, lymphocytes, and eosinophils. The IIR demonstrated an apparent AUC of 0.9711 with an optimal threshold of 7.44 (sensitivity 99.40%, specificity 91.49%). In regression analyses, the IIR emerged as the strongest independent predictor of mortality (adjusted OR 13.98, *p* < 0.001), outperforming established indices such as the neutrophil-to-lymphocyte ratio (NLR) and the cumulative inflammatory index (IIC). Given its simplicity and strong discriminatory power, the IIR may support early risk stratification and prioritization of standard interventions (e.g., intensified monitoring, nutritional support, and timely optimization of anti-TB therapy). Prospective multicenter validation and longitudinal assessment of IIR dynamics are warranted to confirm clinical utility and define applications beyond the index admission, including in resource-limited settings.

## Introduction

Tuberculosis (TB), especially its pulmonary form, and severe malnutrition represent two major public health challenges , whose complex interaction contributes to the amplification of disease burden and increased mortality among vulnerable populations^1^. In countries with limited resources, where access to medical services, early diagnosis, and sustained treatment are problematic, these conditions often overlap, creating an unfavorable epidemiological context. This overlap is especially relevant for pulmonary tuberculosis, which remains the most widespread and severe form of the disease, particularly in resource-limited settings. Socioeconomic factors such as poverty, inadequate nutrition, overcrowding, and poor medical systems not only facilitate the spread of Mycobacterium tuberculosis, but also the onset or exacerbation of malnutrition^2^. Consequently, TB and severe malnutrition reinforce each other, each weakening the defense mechanisms of the body and increasing the risk of unfavorable outcomes and complications^3,4^.

In general, most deaths related to tuberculosis occur in developing countries, where malnutrition remains widespread^5^. In sub-Saharan Africa, the interaction between TB, malnutrition, and HIV co-infection generates some of the most alarming situations, reflecting the complexity of this epidemiological scenario^6^. In West Africa, malnutrition, diagnostic challenges and limited access to appropriate therapies have contributed to the maintenance of high incidence rates of tuberculosis, with Nigeria exemplifying the magnitude of the problem^3^. Furthermore, in Southeast Asia, malnutrition is a critical factor that facilitates the reactivation of latent infections and associates TB with increased mortality rates^7,8^.

Although the situation in Europe does not reach the magnitude observed in Africa or Asia, eastern Europe continues to face a persistent burden of TB, including drug resistant forms (MDR-TB, XDR-TB)^9,10^. In countries such as Romania, Moldova, and Ukraine, the incidence of tuberculosis remains significant, driven by migration, unfavorable socioeconomic factors, and limited access to prevention, diagnostic, and treatment services^11^. In this context, severe malnutrition becomes an amplifier of vulnerability, leading to a decrease in immunological competence and a reduction in the body’s ability to control infection^12^. Although less well-publicized than in other regions, the issue of TB associated with severe malnutrition in eastern Europe needs more attention, as the combination of malnutrition, active TB, and additional comorbidities complicates the prognosis and therapeutic strategies.

At the physiopathological level, malnutrition fundamentally affects immune functions, disrupting the activation and efficiency of T lymphocytes and macrophages, reducing the synthesis of key cytokines (IFN-$\gamma$, TNF-$\alpha$) and thus favoring the rapid progression of the disease^13–15^. Specifically, patients with TB and extremely low BMI have a much higher risk of early mortality, as evidenced by a study conducted in Malawi, where mortality within the first four weeks of treatment was significantly higher among malnourished individuals^16^.

Previous research shows that patients with extremely low BMI suffer from substantially increased mortality, especially in the early weeks of anti-TB therapy^17^. Although indicators such as BMI, C-reactive protein (CRP), and IL-6 have been used to estimate risk and prognosis, their discriminative power is often limited^18–20^. The neutrophil-to-lymphocyte ratio (NLR)^21^, the cumulative inflammatory index (IIC), and mean corpuscular volume of leukocytes (MCVL)^22^, platelet-to-lymphocyte ratio (PLR)^23^, monocyte-to-lymphocyte ratio (MLR)^24^, neutrophil-to-monocyte ratio (NMR)^25^, and lymphocyte-to-monocyte ratio (LMR)^26,27^, have been widely used to predict mortality in cancer, as well as in inflammatory and infectious diseases. However, in pulmonary tuberculosis (PTB) with severe malnutrition, these ratios can misclassify risk: malnutrition is associated with lymphopenia and eosinophil suppression, whereas intercurrent infection and stress responses drive neutrophilia; additionally, fluid shifts and protein depletion further distort composite metrics. These alterations shift the numerators and denominators in directions unrelated to baseline lethality, thereby reducing discriminatory power at presentation. Consequently, a simple and robust biomarker is needed—one that addresses these limitations by jointly reflecting the immune and inflammatory dimensions of PTB in the context of severe malnutrition.

In this study, we propose the Immune-Inflammatory Ratio (IIR)—a data-driven, artificial intelligence–assisted index derived from routine hematologic parameters—to provide a more accurate assessment of mortality risk in patients with PTB and BMI $<16$ kg/m$^{2}$. By integrating key hematologic parameters—including neutrophils, lymphocytes, and eosinophils—the IIR aims to be a straightforward, accessible, and reliable tool, enabling earlier identification of high-risk patients and more refined clinical interventions. Our primary objective was to evaluate the discrimination and calibration of baseline IIR for in-hospital mortality during the index admission; secondary objectives were to define an operating cut-off for bedside triage and to compare IIR with established indices (e.g., NLR, PLR, MLR, NMR).

## Results

### Baseline characteristics of the study population

A total of 216 adult patients diagnosed with tuberculosis (TB) and severe malnutrition (body mass index, BMI $<16$ kg/m$^2$) were included in this analysis, representing 6.09% of the 3,547 TB cases identified during the study period. The mean BMI at baseline was 15.4 ± 2.1 kg/m$^2$. During the course of follow-up, 28 patients (13.0%) died, while 188 (87.0%) survived (Table 1). Clinically, the presence of comorbid conditions (e.g. cardiovascular disease, autoimmune disorders) and systemic symptoms (fever, hemoptysis) was significantly correlated with a higher likelihood of mortality (P<0.001). In addition, productive cough was associated with increased mortality (P=0.004). Overall follow-up during the index admission totalled 13,520 person-days (37.0 person-years), with a median length of stay of 52 days (IQR 35–90).

**Table 1:** Baseline demographic and clinical characteristics of patients by mortality outcome. Sociodemographic and clinical characteristics of patients diagnosed with tuberculosis (TB) and severe malnutrition are stratified by mortality outcome. Data are presented as number (percentage) for categorical variables. P-values derived from Chi-square tests indicate statistical significance for variables such as cardiovascular conditions, asthma, fever, sweating, and hemoptysis. Results highlight the relationship between comorbidities and systemic symptoms with higher mortality risk.

Variable	Level	Deceased (N and %)	Survivors (N and %)	Chi-square	p-value
Sex	Female	8 (22.22%)	28 (77.78%)	2.3717	0.124
	Male	20 (11.11%)	160 (88.89%)		
Environment	Rural	19 (12.58%)	132 (87.42%)	0.0011	0.974
	Urban	9 (13.85%)	56 (86.15%)		
Smoker	Yes	11 (8.87%)	113 (91.13%)	3.5111	0.061
	No	17 (18.48%)	75 (81.52%)		
Alcohol	Yes	8 (9.52%)	76 (90.48%)	0.9853	0.3209
	No	20 (15.15%)	112 (84.85%)		
Asthma	Yes	11 (37.93%)	18 (62.07%)	16.0406	<0.001
	No	17 (9.09%)	170 (90.91%)		
COPD	Yes	6 (17.65%)	28 (82.35%)	0.3693	0.543
	No	22 (12.09%)	160 (87.91%)		
Cardiovascular conditions	Yes	19 (37.25%)	32 (62.75%)	32.1569	<0.001
	No	9 (5.45%)	156 (94.55%)		
Diabetes	Yes	5 (26.32%)	14 (73.68%)	2.1224	0.145
	No	23 (11.68%)	174 (88.32%)		
Productive cough	Yes	24 (18.75%)	104 (81.25%)	8.1093	0.004
	No	4 (4.55%)	84 (95.45%)		
Hemoptysis	Yes	9 (36.00%)	16 (64.00%)	11.0897	<0.001
	No	19 (9.95%)	172 (90.05%)		
Sweating	Yes	24 (37.50%)	40 (62.50%)	17.5354	<0.001
	No	4 (6.92%)	148 (93.08%)		
Fever	Yes	15 (42.86%)	20 (57.14%)	29.9969	<0.001
	No	13 (7.18%)	168 (92.82%)		

### Hematological and biochemical parameters

The comparison between survivors and non-survivors revealed notable differences in hematological and biochemical parameters (Table 2). The mean age of those who died was significantly higher (64.5 vs. 50.0 years, P<0.0001). Hemoglobin (10.78 vs. 12.57 g/dL, P<0.001) and hematocrit levels (32.11% vs. 38.03%, P<0.0001) were lower in the deceased group. Neutrophil counts were significantly elevated (P<0.001) in non-survivors, while lymphocyte (P<0.001) and eosinophil counts (P<0.001) were lower. All leukocyte counts refer to absolute values in $\times$10³/$\mu$L., reflecting an immunologic imbalance associated with a poor prognosis.

**Table 2:** Comparison of age, hematological, and biochemical parameters between survivors and non-survivors. Median values (with interquartile ranges, IQR) for age, hematological, and biochemical parameters are presented for survivors and non-survivors. P-values are derived from the Mann-Whitney U test. Significant differences in hemoglobin, hematocrit, lymphocytes, neutrophils, and eosinophils emphasize the role of anemia and immunologic imbalance in poor clinical outcomes.

Variable	Median deceased (IQR)	Median survivor (IQR)	U statistic	P value
Age (years)	64.50 (58.75, 70.25)	50.00 (43.00, 57.00)	4278.00	<0.001
Hemoglobin (g/dL)	10.78 (9.63, 11.18)	12.57 (11.01, 13.81)	1001.50	<0.001
Hematocrit (fraction)	32.11 (29.68, 34.84)	38.03 (32.91, 41.50)	1213.00	<0.001
Mean corpuscular volume (fL)	83.94 (79.98, 89.45)	86.23 (82.76, 90.56)	2274.50	0.247
Mean hemoglobin (pg)	27.91 (25.66, 29.30)	29.80 (28.05, 30.83)	1566.50	<0.001
Mean corpuscular hemoglobin (g/dL)	32.41 (31.70, 33.99)	33.55 (32.40, 35.10)	1768.00	0.005
Red cell distribution width (units)	16.99 (16.32, 17.53)	15.08 (14.02, 17.08)	3349.50	0.020
Platelets (10$^9$/L)	293.09 (238.58, 385.81)	285.36 (210.41, 337.35)	3035.00	0.192
White blood cells (WBC, 10$^9$/L)	12.25 (10.15, 15.09)	8.61 (6.53, 10.99)	3913.50	<0.001
Lymphocytes (10$^9$/L)	0.80 (0.66, 1.11)	1.60 (1.30, 1.92)	472.50	<0.001
Segmented neutrophils (10$^9$/L)	10.70 (8.81, 13.72)	6.01 (4.21, 8.69)	4365.00	<0.001
Monocytes (10$^9$/L)	0.52 (0.21, 0.92)	0.64 (0.43, 0.81)	2122.00	0.098
Eosinophils (10$^9$/L)	0.02 (0.01, 0.06)	0.12 (0.06, 0.24)	664.00	<0.001
Basophils (10$^9$/L)	0.06 (0.02, 0.09)	0.09 (0.04, 0.12)	1744.00	0.004
Erythrocyte sedimentation rate (mm at 1h)	69.64 (30.60, 93.14)	49.19 (20.00, 89.62)	3193.00	0.069
Erythrocyte sedimentation (mm at 2h)	98.76 (71.49, 117.03)	79.03 (40.32, 109.83)	3259.00	0.042
Aspartate aminotransferase (U/L)	27.04 (11.20, 48.66)	23.23 (18.43, 34.46)	2659.50	0.930
Alanine aminotransferase (U/L)	28.97 (8.14, 32.76)	22.57 (16.91, 34.59)	2692.50	0.846
Direct bilirubin (mg/dL)	0.31 (0.14, 0.43)	0.32 (0.21, 0.40)	2309.50	0.296
Total bilirubin (mg/dL)	0.78 (0.42, 0.96)	0.71 (0.60, 0.90)	2334.00	0.335
Creatinine (mg/dL)	0.77 (0.55, 1.40)	0.85 (0.72, 0.93)	2285.00	0.261
Gamma-glutamyl transferase (U/L)	40.75 (28.10, 69.07)	38.00 (27.58, 55.29)	2960.00	0.289
Triglycerides (mg/dL)	83.25 (68.06, 115.18)	66.41 (55.06, 87.95)	3748.50	<0.001
Uric acid (mg/dL)	40.36 (12.20, 73.92)	18.96 (12.18, 25.00)	3510.00	0.005
Blood glucose (mg/dL)	88.25 (81.08, 100.54)	89.66 (81.86, 116.09)	2213.00	0.175

### Derivation and characterization of the immunoinflammatory ratio (IIR)

**Fig. 1:**
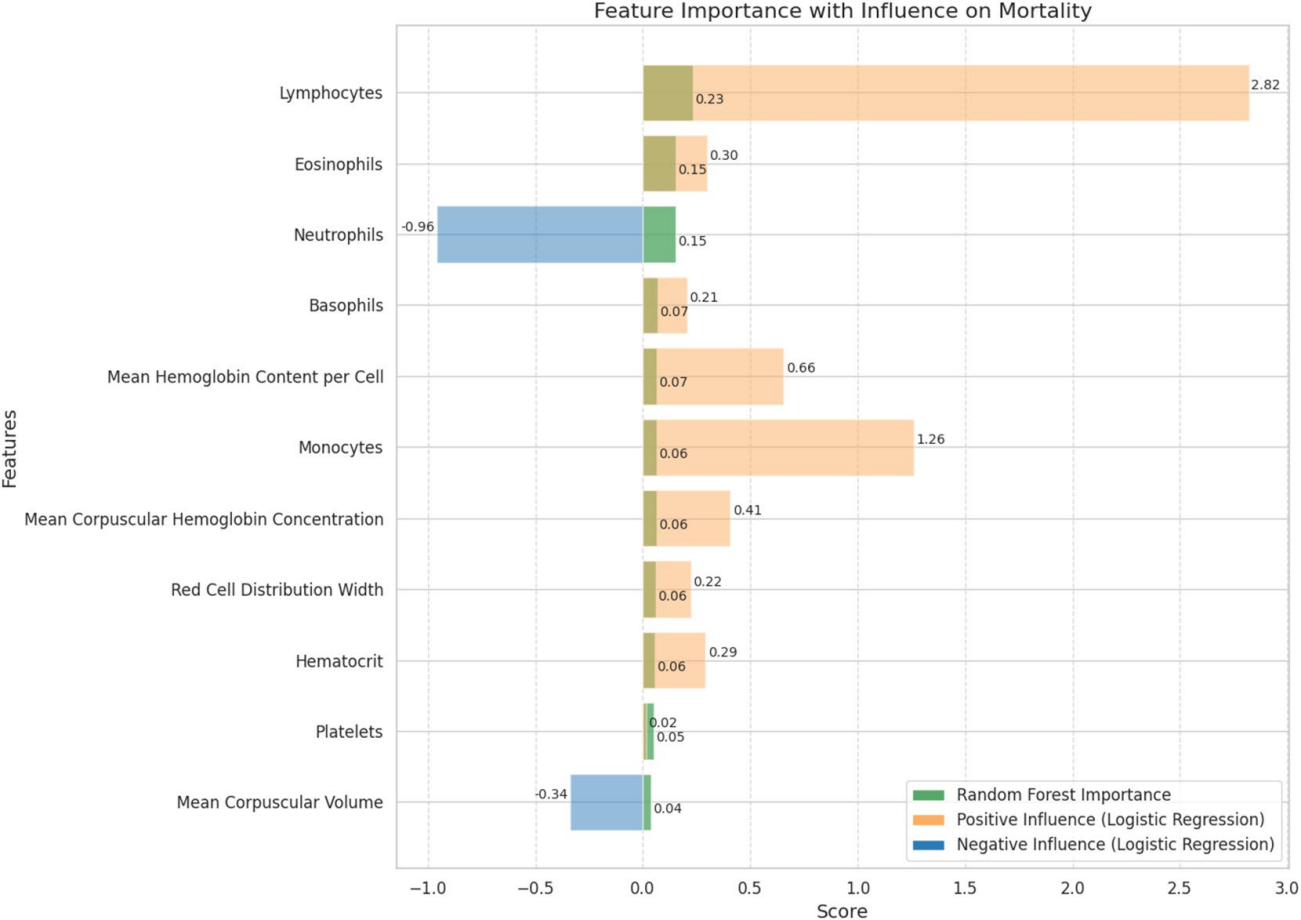
Feature importance analysis for mortality prediction using random forest. The figure illustrates the relative importance of features contributing to mortality prediction in patients with tuberculosis and severe malnutrition. Importance was assessed using the random forest algorithm, with features ranked based on their impact on model performance. Positive and negative influences from logistic regression coefficients are also shown, highlighting the protective effects of lymphocytes and eosinophils, and the negative impact of elevated neutrophil counts.

We derived an immune-inflammatory ratio (IIR) to capture the balance between neutrophils and lymphocytes/eosinophils, defined as (NEU - 1)/(LYM + EOZ) using absolute counts ($\times 10^{3}/\mu \text {L}$). Screening analyses (logistic and random forest; Fig. 1) motivated this structure; full derivation, model coefficients, unit conventions, and worked examples are detailed in Methods.

### Comparison with existing markers and ROC analysis

**Fig. 2:**
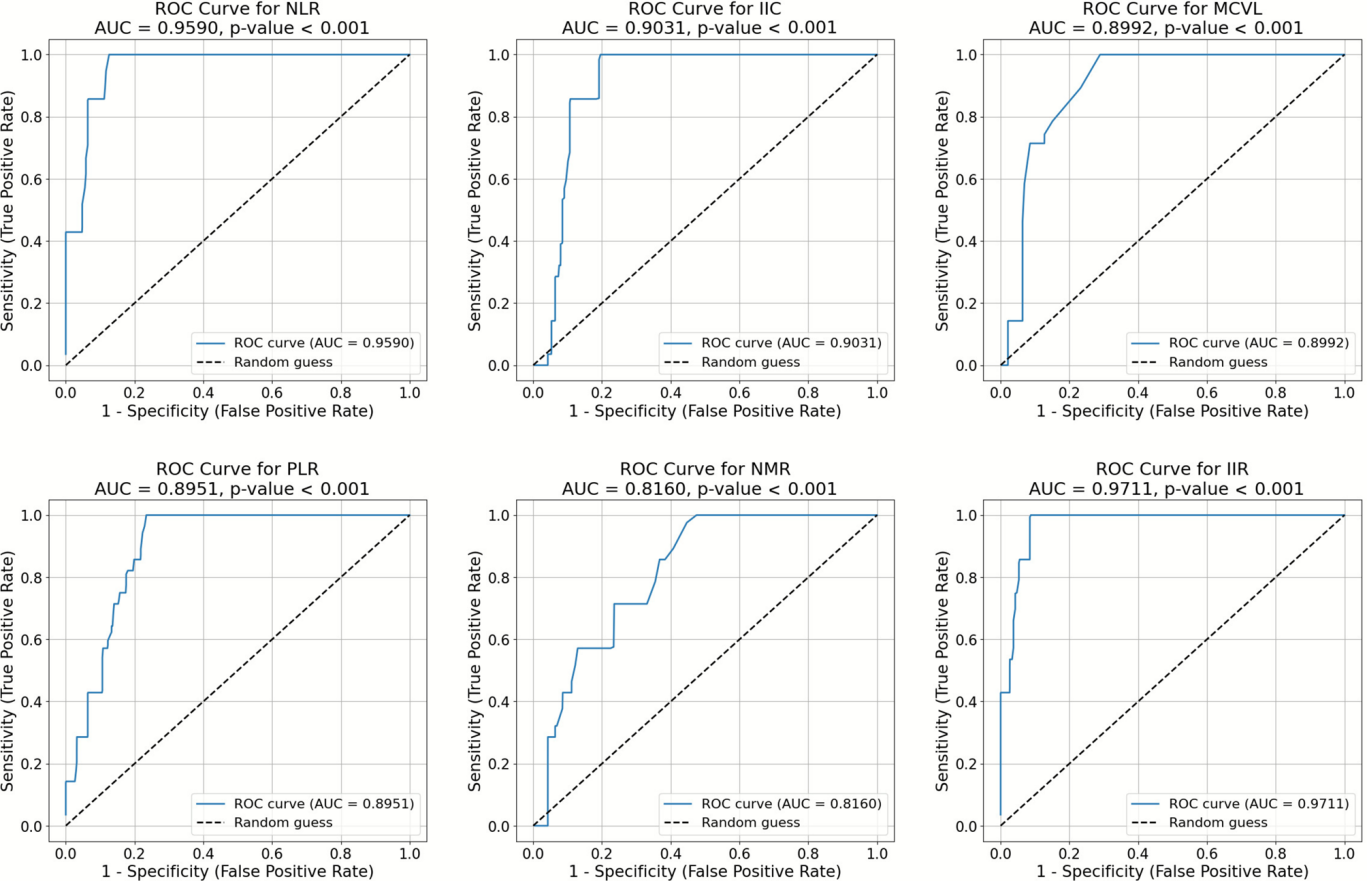
Receiver operating characteristic (ROC) curves for IIR and other markers. ROC curves illustrate the diagnostic performance of IIR and other inflammatory markers, including NLR, IIC, MCVL, PLR, and NMR, in predicting mortality among tuberculosis patients with severe malnutrition. Each curve is associated with an area under the curve (AUC) value, reflecting the discriminative power of the marker. IIR achieved the highest apparent AUC (0.9711), demonstrating superior accuracy compared to other markers.

IIR was compared to other markers derived from the complete blood count, including NLR, IIC and others, to assess its discriminative utility for the stratification of mortality risk in patients with TB with severe malnutrition. Among all markers, IIR showed the most pronounced median difference between survivors and non-survivors, underscoring its stronger association with mortality risk (Table 3). The analysis of the receiver operating characteristic (ROC) curve further demonstrated the superior performance of IIR, achieving an AUC of 0.9711, with a sensitivity of 99.40% and a specificity of 91.49% at an optimal cut-off value of 7.44. Although NLR (AUC = 0.9590) and IIC (AUC = 0.9031) also showed strong performance, IIR had the highest AUC among the indices evaluated (Fig. 2). Internal validation results based on repeated stratified cross-validation with out-of-fold predictions are reported in the “Validation of predictive models” section. Furthermore, markers such as MCVL, PLR, and NMR exhibited lower AUC values, further highlighting the unique potential of IIR as a reliable and integrative biomarker (Table 4).

**Table 3:** Comparison of IIR and other markers between survivors and deceased patients. Data are presented as median values (with interquartile range, IQR) for various hematological and biochemical parameters, such as age, hemoglobin, white blood cells, and liver enzymes, comparing survivors and non-survivors. The U statistics and p-values are shown to highlight statistically significant differences.

Marker	Median deceased (IQR)	Median survivor (IQR)	U statistic	p-value
NLR	11.57 (11.12, 20.79)	3.61 (2.35, 5.14)	5048.00	<0.001
IIC	17.51 (16.19, 26.10)	5.05 (3.56, 7.17)	4754.00	<0.001
MCVL	102.81 (73.80, 116.33)	52.07 (42.94, 69.29)	4733.50	<0.001
PLR	359.34 (301.97, 593.85)	174.59 (130.14, 240.48)	4712.00	<0.001
LMR	1.46 (1.07, 2.39)	2.92 (1.74, 4.03)	1437.50	<0.001
MLR	0.68 (0.42, 0.94)	0.34 (0.25, 0.57)	3823.00	<0.001
NMR	22.64 (12.61, 49.41)	9.05 (5.73, 15.68)	4295.50	<0.001
IIR	10.04 (9.46, 18.99)	2.78 (1.47, 3.98)	5112.00	<0.001

**Table 4:** ROC curve analysis of IIR and established markers for predicting mortality. Area under the curve (AUC), cut-off values, sensitivity, specificity, and P-values for markers such as NLR, IIC, MCVL, PLR, and IIR are presented. IIR exhibited the highest apparent AUC (0.9711), with optimal sensitivity and specificity, demonstrating superior performance in predicting mortality risk. These findings highlight IIR’s potential as a robust clinical tool for risk stratification.

Marker	AUC	Cut-off value	Sensibility	Specificity	p-value
NLR	0.9590	8.30	94.72	88.30	<0.001
IIC	0.9031	9.24	98.39	80.85	<0.001
MCVL	0.8992	69.60	89.29	76.72	<0.001
PLR	0.8951	255.90	96.43	77.08	<0.001
LMR	0.2731	1.14	64.69	0.06	<0.001
MLR	0.7236	0.16	98.41	10.64	0.006
NMR	0.8160	10.18	97.59	55.32	<0.001
IIR	0.9711	7.44	99.40	91.49	<0.001

### Univariable and multivariable risk factor analysis

**Fig. 3:**
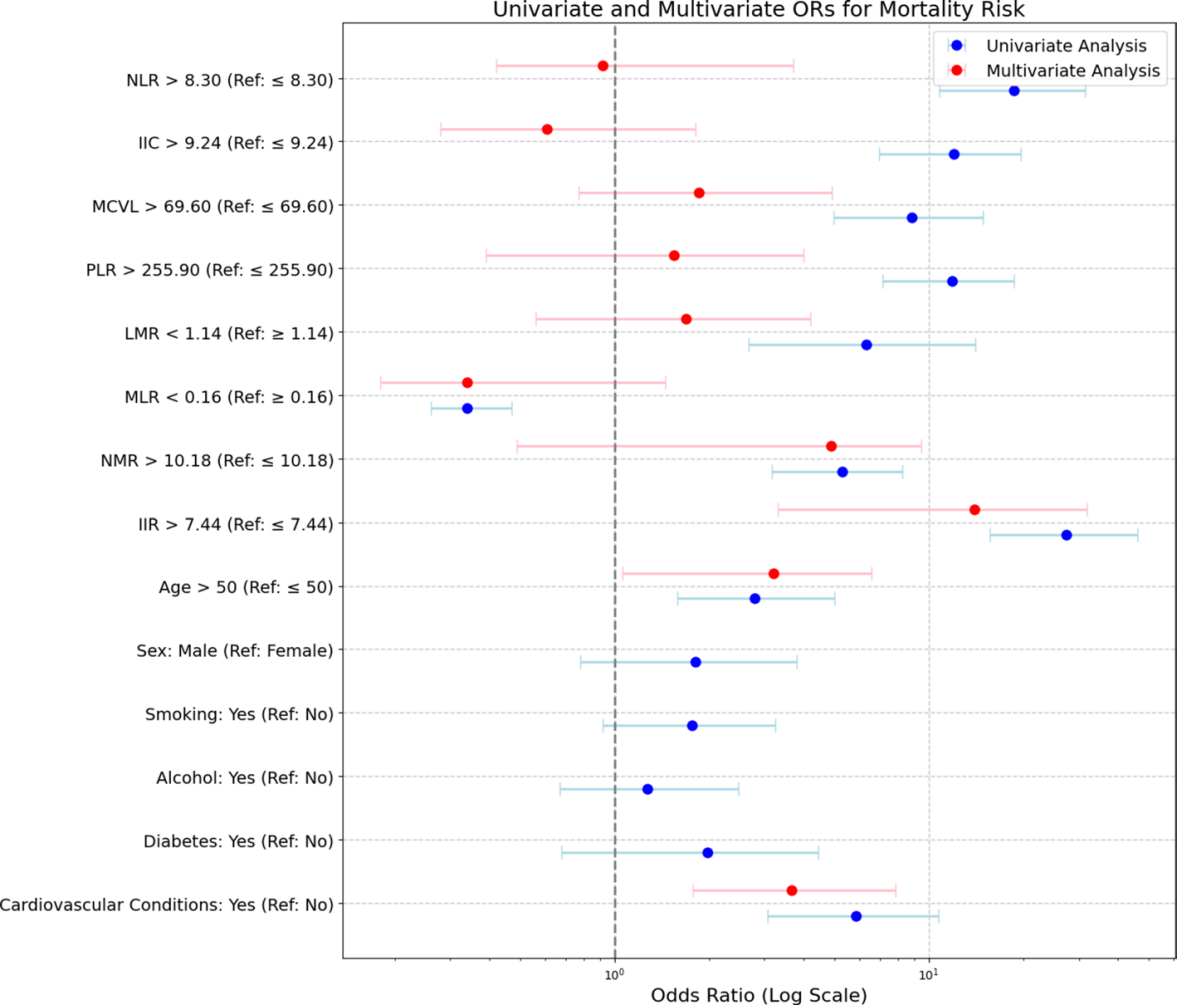
Forest plot of univariate and multivariate odds ratios for mortality risk. The forest plot displays odds ratios (OR) with 95% confidence intervals (CI) for univariate and multivariate analyses of various mortality risk factors. Key variables include IIR, NLR, MCVL, and comorbid conditions such as cardiovascular diseases. Multivariate adjustments account for confounders like age and sex. The plot highlights the strong predictive value of IIR (OR=13.98) and cardiovascular conditions (OR=3.66), emphasizing their independent association with mortality risk.

**Fig. 4:**
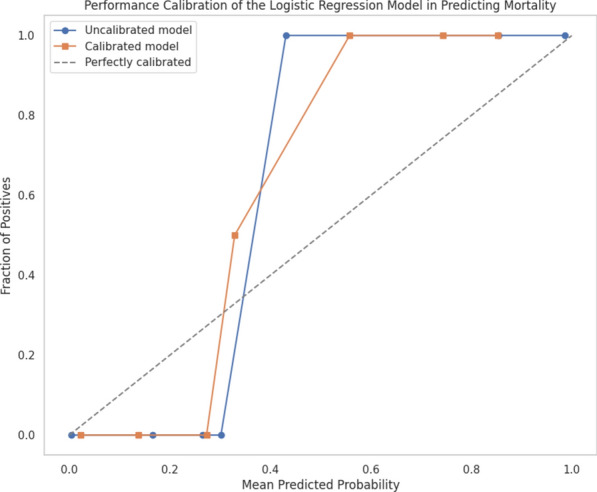
Logistic regression calibration performance. Calibration performance of the logistic regression model for predicting mortality in tuberculosis patients with severe malnutrition is illustrated. The plot compares predicted probabilities with observed mortality rates. The uncalibrated model aligns more closely with the ideal diagonal (perfect calibration), while the calibrated model shows slight deviations, indicating a minor reduction in predictive accuracy after calibration.

In the univariate analysis, IIR showed an odds ratio (OR) of 27.39 (P<0.001), underscoring the strong association between elevated IIR values and mortality (Table 5). NLR, which showed a significant association in the univariate analysis (OR=18.68, P<0.001), lost its predictive power in the multivariable model (OR=0.92, P=0.887), suggesting that its effect was confounded by other factors included in the model (Table 5). In contrast, IIR remained the most powerful independent predictor of mortality in multivariate models adjusted for variables such as age, sex, cardiovascular disease, smoking status and alcohol use (OR=13.98, P<0.0001) (Fig. 3). The logistic model showed excellent calibration (Brier Score=0.0138), correctly classifying the vast majority of fatal outcomes (Fig. 4). Additional calibration via Platt Scaling slightly increased the Brier Score to 0.0270.

**Table 5:** Univariate and multivariate analysis of mortality risk factors. Univariate and multivariate odds ratios (OR) with 95% confidence intervals (CI) and P-values for various risk factors such as NLR, IIC, PLR, and IIR are presented. The multivariate model, adjusted for age, sex, and cardiovascular conditions, shows the strong independent predictive power of IIR (OR=13.98, P<0.0001). Cardiovascular conditions and age >50 are also significant predictors, underscoring the role of systemic inflammation and comorbidities in mortality risk.

Variable	Univariate OR (95% CI)		Multivariate OR (95% CI)	
	OR	p-value	OR	p-value
NLR > 8.30 (Ref: $\le$ 8.30)	18.68 (10.81–31.36)	<0.001	0.92 (0.42–3.71)	0.887
IIC > 9.24 (Ref: $\le$ 9.24)	12.00 (6.94–19.66)	<0.001	0.61 (0.28–1.81)	0.317
MCVL > 69.60 (Ref: $\le$ 69.60)	8.80 (4.99–14.90)	<0.001	1.85 (0.77–4.91)	0.193
PLR > 255.90 (Ref: $\le$ 255.90)	11.85 (7.11–18.63)	<0.001	1.55 (0.39–3.99)	0.419
LMR < 1.14 (Ref: $\ge$ 1.14)	6.31 (2.67–14.06)	<0.001	1.69 (0.56–4.19)	0.320
MLR < 0.16 (Ref: $\ge$ 0.16)	0.34 (0.26–0.47)	<0.001	0.34 (0.18–1.45)	0.050
NMR > 10.18 (Ref: $\le$ 10.18)	5.30 (3.16–8.24)	<0.001	4.89 (0.49–9.46)	0.037
IIR > 7.44 (Ref: $\le$ 7.44)	27.39 (15.61–46.10)	<0.001	13.98 (3.31–31.87)	<0.001
Age > 50 (Ref: $\le$ 50)	2.80 (1.59–5.00)	<0.001	3.20 (1.06–6.57)	0.008
Sex: Male (Ref: Female)	1.81 (0.78–3.81)	0.142		
Smoking: Yes (Ref: No)	1.76 (0.92–3.24)	0.078		
Alcohol: Yes (Ref: No)	1.27 (0.67–2.47)	0.470		
Diabetes: Yes (Ref: No)	1.97 (0.68–4.45)	0.157		
Cardiovascular Conditions: Yes (Ref: No)	5.86 (3.06–10.75)	<0.001	3.66 (1.77–7.83)	<0.001

### Validation of predictive models

**Fig. 5:**
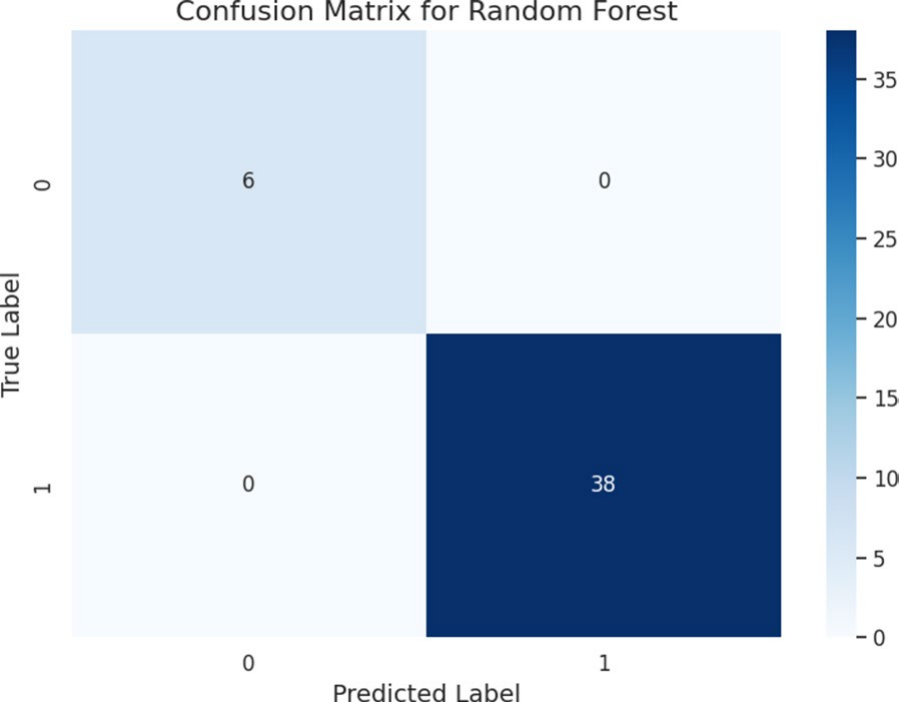
Performance evaluation of Random Forest using confusion matrix. The matrix shows the number of correctly and incorrectly classified cases for survival and in-hospital death. The model correctly classified 38 survivors and 6 deaths, with no misclassifications in the test set. The positive class was defined as survival, following the original DECES coding in the source dataset.

**Fig. 6:**
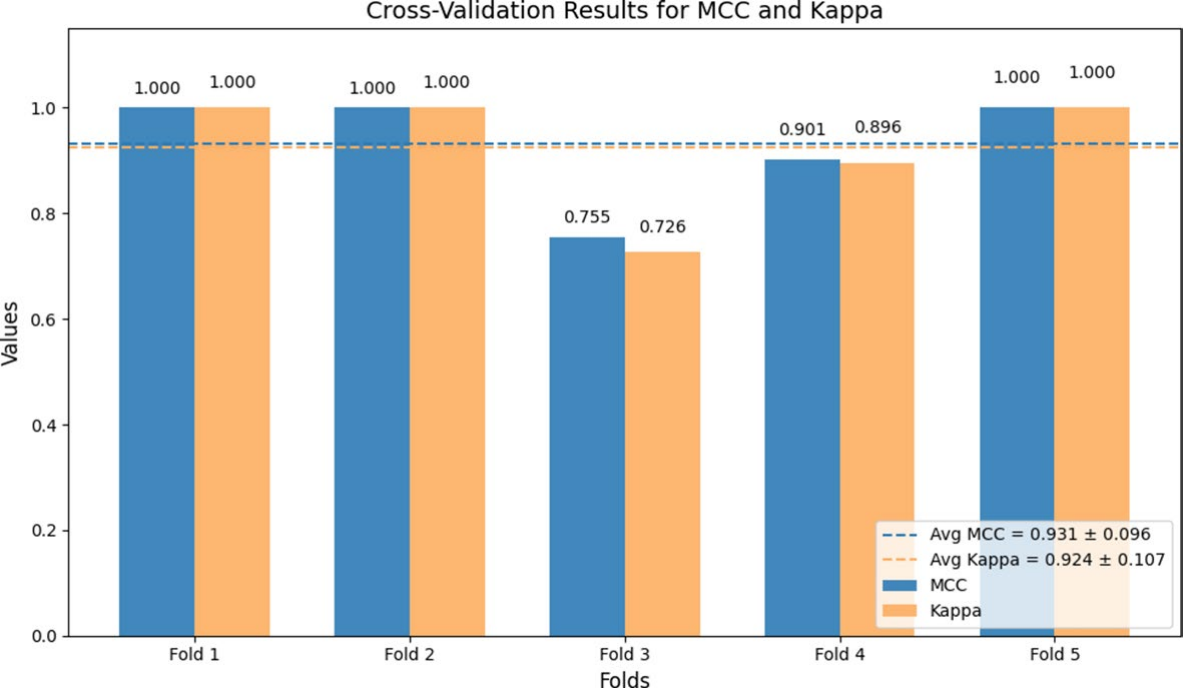
Performance metrics in cross-validation: MCC and Kappa scores. Cross-validation results for the predictive model are shown, highlighting the Matthews Correlation Coefficient (MCC) and Cohen’s Kappa scores across five folds. The model achieves an average MCC of 0.931 ± 0.096 and an average Kappa score of 0.924 ± 0.107, reflecting high accuracy and reliability. Consistent performance across folds demonstrates the robustness and generalizability of the model, with only minor variations observed in Fold 3.

**Fig. 7:**
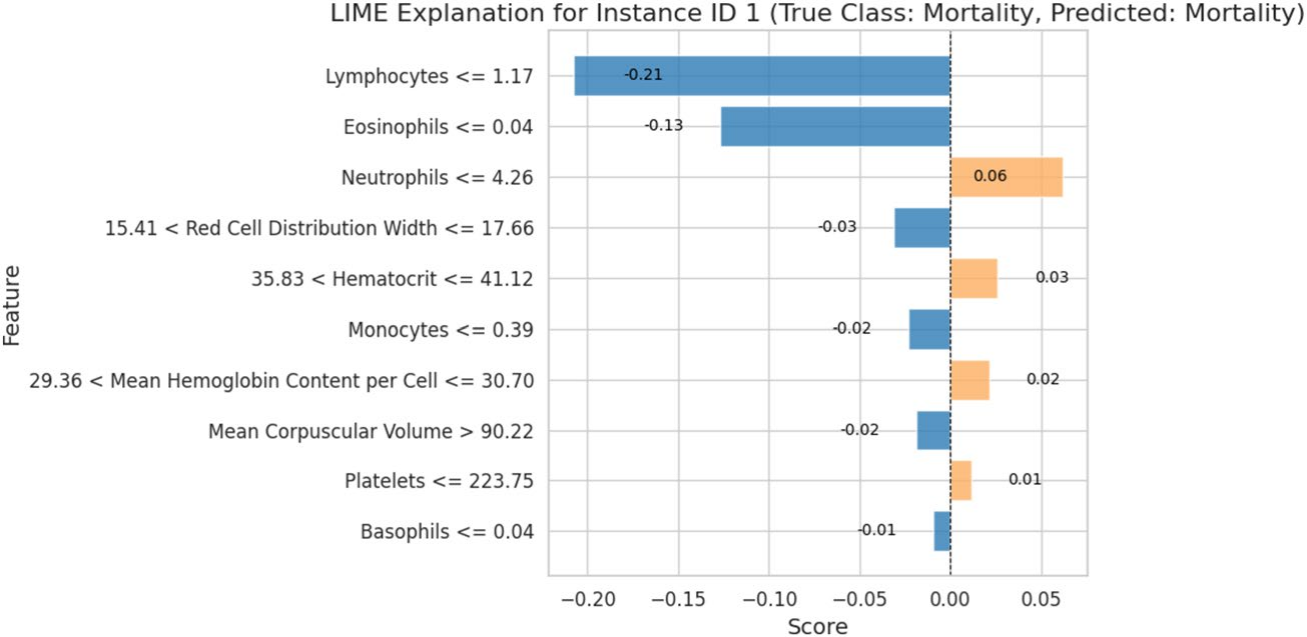
Key predictors of mortality identified by LIME. Local Interpretable Model-Agnostic Explanations (LIME) analysis highlights the most influential features contributing to mortality prediction for a specific instance. Key variables include lymphocyte and eosinophil counts (negative impact) and neutrophil levels (positive impact). The figure provides a detailed breakdown of feature contributions, enabling better understanding of the model’s decision-making process and emphasizing the biological relevance of these predictors in the context of tuberculosis and severe malnutrition.

To ensure the stability and reliability of our predictive models, we employed rigorous cross-validation methods. The performance metrics derived from the Random Forest algorithm were outstanding, with a confusion matrix showing perfect accuracy, correctly classifying all cases in the test set (Fig. 5). The consistency of these models across multiple cross-validation folds was summarized by Matthew’s Correlation Coefficient (MCC) = $0.931 \pm 0.096$ and Cohen’s Kappa = $0.924 \pm 0.107$. (Fig. 6). Local Interpretable Model-Agnostic Explanations (LIME) indicated that variations in lymphocyte, eosinophil, and neutrophil counts were key contributors (Fig. 7). To mitigate optimism, we assessed internally validated discrimination for IIR using repeated stratified cross-validation with out-of-fold predictions. The resulting out-of-fold AUC was 0.882 (bootstrap 95% CI 0.798–0.942).

## Discussion

Our findings offer a deeper understanding of the complex interactions between TB, severe malnutrition, and the immune system of the host. By introducing the IIR, we aimed to provide a practical but robust single metric that consolidates both pro-inflammatory components and protective elements. This approach aims to overcome the limitations of conventional markers by providing a broader perspective on the immune-inflammatory environment. In fact, our analyses suggest that IIR can distinguish patients with elevated mortality risk from those with comparatively more favorable outcomes, underscoring the potential benefit of modeling the immune-inflammatory response in this way, particularly in the context of TB coupled with severe malnutrition.

A defining feature of IIR is the one-unit adjustment applied to neutrophil count, mirroring the initial logistic analyzes, which indicated a robust negative impact of neutrophils on the clinical prognosis^28–30^. Typically, neutrophils serve as a first-line defense by engaging in phagocytosis, generating reactive oxygen species, and releasing proteolytic enzymes^29,31,32^. However, in TB, especially under resource-deprived conditions such as severe malnutrition, an excess of neutrophils can intensify lung tissue damage. Furthermore, malnutrition disrupts neutrophil migration and affects their effector functions^33–36^, potentially creating a hyperinflammatory environment that is more difficult to control when the body lacks adequate nutritional reserves.

In contrast, lymphocytes and eosinophils demonstrated protective associations. T lymphocytes are central to adaptive immunity, helping eradicate Mycobacterium tuberculosis by activating macrophages and orchestrating cytokine release^37–39^. B lymphocytes and antibody-mediated responses further support persistent immunity. However, in the context of severe malnutrition, lymphocyte counts often drop significantly, compromising the adaptive immune response.^40–44^. Consequently, undernourished patients become more susceptible to severe or protracted infections. Although eosinophils traditionally have been linked to allergic and antiparasitic responses, they also help modulate inflammation^45–47^. In states of eosinopenia, the body’s ability to combat excessive inflammation can be compromised^48–52^.

Malnutrition also affects the immune system by suppressing immune cell production, disrupting cytokine synthesis, and weakening mucosal barriers.^53,54^. Studies indicate that low-calorie intake can alter immune coordination, lowering essential cytokines (e.g., IFN-$\gamma$, IL-12) and disturb the balance among immune cell subsets^54–56^. Undernutrition can also facilitate the translocation of bacteria from the gut to the bloodstream, increasing the risk of secondary infections^57^. This malnutrition–inflammation loop has been recognized in other clinical syndromes, including the malnutrition–inflammation axis observed in chronic renal failure, where it correlates with poorer survival^58–62^.

In TB, an unbridled inflammatory response, especially if not backed by a robust adaptive immune system, may accelerate disease progression, encourage extensive tissue damage, and contribute to multi-organ dysfunction^33,63–66^. Severe malnutrition only magnifies these impacts by depleting the physiological reserves and resilience of the body. As a result, a vicious cycle emerges: malnutrition alters immunity, facilitates infection, and exacerbates inflammation; in turn, chronic inflammation and increased metabolic demands further sustain the state of undernutrition^67–69^. In the absence of timely, tailored interventions, this cycle becomes self-reinforcing, increasing the likelihood of mortality.

IIR provides an attempt to integrate these overlapping mechanisms into a low-invasive and cost-effective tool that can be used in everyday clinical practice for early risk stratification. Compared to existing markers, IIR showed superior discriminative performance, suggesting that integrating immunologic and inflammatory data in a single parameter could yield meaningful benefits. Importantly, this does not replace standard care: all severely underweight pulmonary TB (PTB) patients should receive prompt nutritional support and guideline-concordant anti-TB therapy. The role of IIR–particularly values above the operating threshold of 7.44 in this cohort–is to help prioritize the timing and intensity of these same interventions when resources or capacity are constrained. In practical terms, patients with very high IIR at admission may benefit from an ”escalation bundle” during the index hospitalization: early senior review; closer monitoring or step-up care; protocolized refeeding with electrolyte surveillance to prevent refeeding syndrome; rapid correction of dehydration and anemia; screening and early treatment of intercurrent infections; and expedited microbiology, including rapid drug-susceptibility testing to minimize time to effective therapy. Discharge planning for this subgroup should include structured early follow-up (e.g., within 7 days), adherence support, and linkage to social services when relevant. These components extend, rather than replace, standard care and remain feasible in settings without access to advanced biomarkers. Given its simplicity of calculation and superior predictive performance, IIR has the potential to be integrated into clinical guidelines for tuberculosis management, particularly in resource-limited settings. By allowing earlier identification of high-risk patients, IIR could inform the prioritization of nutritional support and optimization of anti-TB therapy, ultimately improving clinical outcomes and reducing mortality. Prospective studies should evaluate whether IIR-guided triage (e.g., using a threshold >7.44) improves difficult outcomes, including in-hospital and post-discharge mortality, nutritional recovery, and time to effective therapy.

Our study has several limitations. Mortality data was limited to the initial hospitalization period, and baseline analyzes were performed using data collected before the initiation of any therapy. Although this approach minimizes the potential confounding effects of treatment, it does not account for late-stage complications or the results of subsequent interventions. Furthermore, we did not consider socioeconomic and other confounding factors (e.g., undiagnosed chronic conditions, co-infections, or healthcare access issues) that could also influence inflammatory responses. In addition, our cohort was drawn from a single center, raising questions about external validity that must be addressed through additional multicenter investigations. Follow-up beyond index admission was not captured; therefore, the clinical implications discussed here relate specifically to in-hospital risk stratification. Future studies should focus on the longitudinal evaluation of the dynamics of IIR throughout anti-TB treatment to determine its utility in monitoring disease progression and response to therapy. Such investigations could involve repeated IIR measurements at predefined intervals to identify critical thresholds for treatment adjustment. Including IIR in a national protocol can further highlight its dynamic profile and clarify the clinical utility of this novel marker.

## Methods

### Study population and study design

This ambispective 3-year study (1 October 2021–30 September 2024) was conducted at Leamna Hospital, a regional reference center for the diagnosis and management of TB in Oltenia, Romania. The ambispective approach enabled both retrospective data collection and prospective follow-up of patient clinical courses. We included adult patients ($\ge$18 years) with confirmed pulmonary tuberculosis (smear microscopy, Lowenstein-Jensen culture and/or GeneXpert MTB/RIF) and severe malnutrition (body mass index, BMI <16 kg/m²). If a patient had multiple hospitalizations, only index admission was considered; subsequent admissions were excluded from all analyses. Follow-up accrued as person-time from admission to discharge during index hospitalization, and cohort follow-up was summarized in person-days and person-years. Exclusion criteria included the presence of known autoimmune diseases, recent acute infections (within the last 3 months), severe chronic comorbidities (e.g., advanced renal failure, active neoplasms), ongoing immunosuppressive therapy, pregnancy, and lack of complete datasets or inability to participate in scheduled assessments. Out of the 3547 pulmonary tuberculosis patients evaluated over the study period, 216 (6.09%) met the eligibility criteria and were ultimately included in the analysis. The study was approved by the Ethics Committee of Leamna Hospital and written informed consent was obtained from all participants. The approval numbers were 6109 (18 February 2022) for the two-year prospective component and 6104 (28 August 2024) for the one-year retrospective interval. All procedures were performed in accordance with the Declaration of Helsinki.

### Data collection

For each participant, demographic (age, sex) and clinical data (symptoms, comorbidities) as well as lifestyle information (smoking status, alcohol consumption) were collected. Hematologic parameters (e.g., neutrophil, lymphocyte, and eosinophil counts) and biochemical measures (e.g., hemoglobin, hematocrit) were obtained from existing medical records and supplemented by structured interviews at admission. TB diagnoses were based on sputum smear microscopy (acid-fast bacilli), Lowenstein-Jensen culture, and GeneXpert MTB / RIF tests to detect resistance to isoniazid and rifampicin. Patients were monitored until the end of the follow-up period to document mortality status. Mortality was assessed exclusively during the initial hospitalization period, and subsequent admissions were excluded. All hematological and biochemical parameters included in the analysis were collected at baseline, prior to the initiation of anti-TB therapy or any other medical intervention. All leukocyte counts (NEU, LYM, EOZ) were handled as absolute values in $\times 10^{3}/\upmu \text {L}$ (numerically identical to $\times 10^{9}/\text {L}$).

### Modeling framework and IIR derivation

#### Modeling framework (logistic equation with coefficients)

In-hospital mortality was modeled via logistic regression with IIR as the predictor:

$$\text {logit}\{p(\text {in-hospital death})\} = \beta _{0} + \beta _{1} \times \text {IIR}, \qquad p = \frac{1}{1 + \exp [-(\beta _{0} + \beta _{1} \cdot \text {IIR})]}.$$

Estimated coefficients for this cohort were: Intercept ($\beta _{0}$) = $-7.528$ (95% CI: $-10.211$ to $-4.846$; $p < 0.001$) and IIR ($\beta _{1}$) = 0.770 (95% CI: 0.468 to 1.073; $p < 0.001$).

#### Units

NEU, LYM, and EOZ were handled as absolute counts expressed in $\times 10^{3}/\mu \text {L}$ (numerically identical to $\times 10^{9}/\text {L}$); for example, 7,000 cells/$\mu \text {L}$ is equivalent to $7.0 \times 10^{3}/\mu \text {L}$. All IIR computations and thresholds used these units.

#### Derivation and motivation

To identify the most relevant hematologic factors for the prediction of mortality, we used both logistic regression and random forest algorithms (Figure 1). The variable importance analysis revealed a strong protective effect of lymphocytes (logistic coefficient  +2.82) and eosinophils (+0.30), in contrast to the negative association observed for neutrophils (-0.96). Based on these findings, we developed the immuno-inflammatory ratio (IIR), defined as

$$\text {IIR} = \frac{(\text {Neutrophils} - 1)}{(\text {Lymphocytes} + \text {Eosinophils})}$$

Why this formulation?

- (Neutrophils - 1): The logistic coefficient for neutrophils ( -0.96, close to -1) indicated the need for an adjustment. Subtracting ’1’ from the neutrophil count normalizes their pro-inflammatory influence, aligning risk-based values on a more interpretable scale. Without this correction, the negative impact of neutrophils could be underestimated or difficult to interpret in a composite index such as the IIR.
- (Lymphocytes + Eosinophils): Both lymphocytes and eosinophils emerged as protective factors. Summing these cell types within the denominator amplifies their combined ”anti-inflammatory” effect. Incorporating both into the same denominator allows IIR to capture the balance between pro-inflammatory and protective elements in a straightforward and biologically relevant manner.

The selection of this formula was guided not only by logistic coefficients, but also by iterative testing in predictive models to ensure that IIR remains easily calculable and interpretable in clinical practice while conveying robust and biologically pertinent information. **Worked examples (IIR computation).** **Vignette 1 (higher risk).** NEU = 9.2, LYM = 0.8, EOZ = 0.0 ($\times 10^{3}/\mu \text {L}$)

$$\text {IIR} = \frac{9.2 - 1}{0.8 + 0.0} = 10.25.$$

#### Vignette 2 (lower risk)

NEU = 5.0, LYM = 1.6, EOZ = 0.2 ($\times 10^{3}/\mu \text {L}$)

$$\text {IIR} = \frac{5.0 - 1}{1.6 + 0.2} = 2.22.$$

### Statistical analysis

All data processing and statistical analyzes were performed exclusively in Python 3.9.13, using specialized libraries such as pandas, numpy, scikit-learn, matplotlib and seaborn. Variable distributions were assessed using the Shapiro-Wilk test. Comparisons between survivors and non-survivors employed the Mann-Whitney U test for continuous variables and the chi-square test for categorical variables. For 2$\times$2 tables with any expected cell count < 5, Fisher’s exact test was used. P-values are reported to three decimals; values < 0.001 are presented as $p < 0.001$ (two-sided $\alpha = 0.05$). To derive and validate the IIR, the data set was randomly split into a straified training cohort (80%) and a test cohort (20%). Model optimization and validation were performed through cross-validation procedures. We employed algorithms such as Random Forest and Decision Tree, evaluated using receiver operating characteristic (ROC) curves and area under the ROC curve (AUC) calculations and used these models for feature-importance screening alongside logistic regression. For enhanced interpretability and to pinpoint key variables, we applied feature importance assessments and LIME (Local Interpretable Model-Agnostic Explanations). The final results were summarized through ROC curves, tables detailing AUC values, sensitivity, and specificity, as well as univariate and multivariate logistic regression analyzes. In the source database, the Romanian variable DECES is encoded as 0 = death and 1 = survival. For regression analyses, we created DECES_INV = $1 - \texttt {DECES}$ so that 1 indicates the event (in-hospital death), consistent with standard logistic regression conventions.

#### Statistical modeling and validation (procedures)

##### Univariate logistic regression

Separate logistic models were fitted for each candidate predictor with outcome in-hospital death. We report odds ratios (ORs) with 95% confidence intervals and p-values to three decimal places; values < 0.001 are presented as $p < 0.001$.

##### Multivariable model building and variable selection

The multivariable logistic model was prespecified to include IIR and clinically relevant covariates (age, sex, cardiovascular disease, smoking, alcohol) based on a priori knowledge from the TB/malnutrition–inflammation literature. No automated stepwise procedures were used. Additional predictors were considered if they showed univariate association at $p < 0.20$, improved AIC/BIC, and did not introduce problematic collinearity (variance inflation factor, VIF > 5).

##### Confounding assessment

Potential confounders were identified *a priori* (same references as above) and evaluated using change-in-estimate criteria; variables producing a $\ge 10\%$ change in the IIR coefficient were retained regardless of statistical significance. Collinearity was assessed using VIFs and correlation matrices. Where constructs overlapped (e.g., IIR versus other CBC ratios), the prespecified primary predictor (IIR) was retained for parsimony and interpretability.

##### Regularization

L2-penalized (ridge) logistic regression was evaluated to mitigate overfitting and collinearity. Penalty strength (*C*) was tuned via stratified 5-fold cross-validation on the training set (criterion: mean log-loss), then refit on the full training data and evaluated on the test set.

##### ROC analysis and threshold selection

Discrimination was quantified using AUC with bootstrap 95% confidence intervals (1,000 resamples). Sensitivity, specificity, PPV, and NPV were reported at the operating threshold selected by Youden’s *J* on the training set and evaluated on the test set.

##### Calibration

Calibration metrics included the Brier score, calibration intercept and slope, and calibration curves (loess-smoothed). Platt scaling (logistic recalibration) was explored within validation folds; primary reporting uses the unscaled logistic model unless otherwise stated.

##### Data splits

A single random split was used: 80% training and 20% test. Test-set metrics are fully out-of-sample with respect to tuning and threshold selection. Sample size and statistical justification. This was an exhaustive cohort over the study window. The primary objective focused on a parsimonious logistic model with a single prespecified predictor (IIR). With $N = 216$ and 28 events (12.96%), the primary model satisfies standard events-per-variable considerations, and the IIR effect estimate demonstrated acceptable precision ($\beta _{1} = 0.770$, SE = 0.154; 95% CI 0.468–1.073; $p < 0.001$). For discrimination, an AUC-based power calculation (two-sided $\alpha = 0.05$, power = 0.80, $H_{0}$ AUC = 0.50) indicated a minimum required sample size of approximately $N \approx 43$ ($\approx 6$ events and $\approx 37$ non-events); the available sample ($N = 216$) exceeded this requirement. To limit overfitting in adjusted analyses, degrees of freedom were constrained a priori (age, sex, cardiovascular disease, smoking, alcohol), and internal validation (repeated stratified cross-validation with out-of-fold predictions, bootstrap, and L2 regularization) was used to quantify optimism in discrimination and calibration.

## Conclusions

The present study underscores the potential of the Immuno-Inflammatory Ratio (IIR) as an integrative, admission-time biomarker for stratifying in-hospital mortality risk in patients with pulmonary TB and severe malnutrition. By combining neutrophils, lymphocytes, and eosinophils into a straightforward index, the IIR addresses limitations of conventional markers such as NLR and IIC, offering a more refined view of immune–inflammatory dysregulation. While all severely underweight PTB patients require guideline-concordant care, IIR values above  7.44 may serve as a triage signal to escalate the timing and intensity of standard interventions–early dietitian review; protocolized refeeding with thiamine and close electrolyte monitoring; high-protein, energy-dense supplementation with escalation to enteral feeding if needed; rapid correction of dehydration and anemia; expedited microbiology to minimize time to an effective regimen; and strengthened adherence and laboratory surveillance. These steps augment rather than replace usual care. External, multicenter validation and longitudinal assessment of IIR dynamics remain essential to confirm clinical applicability. If validated, the IIR could support pragmatic bedside prioritization and contribute to a new standard for risk stratification in TB management, particularly in resource-constrained settings.

## Data Availability

The research data supporting the findings of this study are not publicly available due to participant confidentiality and GDPR regulations. The informed consent obtained from participants did not include permission for public disclosure of raw data. However, anonymized data can be made available upon reasonable request and under conditions that ensure compliance with GDPR and ethical standards. Interested researchers may contact Emil-Tiberius Trașcă (etrasca@yahoo.com) or Patricia-Mihaela Rădulescu (patricia.radulescu@umfcv.ro) for further information.

## Abreviations

AUC: Area under the curve
BMI: Body mass index
CRP: C-reactive protein
GeneXpert: MTB/RIF GeneXpert Mycobacterium Tuberculosis/Rifampicin
IFN-$\gamma$: Interferon gamma
IIC: Cumulative inflammatory Index
IIR: Immuno-inflammatory ratio
IL-12: Interleukin-12
MCC: Matthew’s correlation coefficient
MCVL: Mean corpuscular volume of lymphocytes
MLR: Monocyte-to-lymphocyte ratio
NLR: Neutrophil-to-lymphocyte ratio
NMR: Neutrophil-to-monocyte ratio
PLR: Platelet-to-lymphocyte ratio
ROC: Receiver operating characteristic
TB: Tuberculosis

## Code availability

The code central to the conclusions, together with analysis scripts and a documented synthetic dataset (reproducibility package), is publicly available on Zenodo (record: 18042276; DOI: 10.5281/zenodo.18042276)^70^.
